# Antioxidant Status of Broiler Chickens Fed Diets Supplemented with Vinification By-Products: A Valorization Approach

**DOI:** 10.3390/antiox10081250

**Published:** 2021-08-05

**Authors:** Alexandros Mavrommatis, Elisavet Giamouri, Eleni D. Myrtsi, Epameinondas Evergetis, Katiana Filippi, Harris Papapostolou, Sofia D. Koulocheri, Evangelos Zoidis, Athanasios C. Pappas, Apostolis Koutinas, Serkos A. Haroutounian, Eleni Tsiplakou

**Affiliations:** 1 Laboratory of Nutritional Physiology and Feeding, Department of Animal Science, School of Animal Biosciences, Agricultural University of Athens, Iera Odos 75, GR-11855 Athens, Greece; mavrommatis@aua.gr (A.M.); egiamouri@aua.gr (E.G.); elenamirtsi@aua.gr (E.D.M.); epaev@aua.gr (E.E.); skoul@aua.gr (S.D.K.); ezoidis@aua.gr (E.Z.); apappas@aua.gr (A.C.P.); sehar@aua.gr (S.A.H.); 2 Laboratory of Food Process Engineering, Department of Food Science and Human Nutrition, Agricultural University of Athens, Iera Odos 75, GR-11855 Athens, Greece; filippi@aua.gr (K.F.); harris_papapostolou@yahoo.gr (H.P.); akoutinas@aua.gr (A.K.)

**Keywords:** grape pomace, grape stems, polyphenols, liver, wine yeast cells, wine lees, flavonoids

## Abstract

Vinification by-products display great potential for utilization as feed additives rich in antioxidant compounds. Thus, the effect of dietary ground grape pomace (GGP), wine lees extract rich in yeast cell walls (WYC), and grape stem extracts (PE) on the relative expression of several genes involved in liver oxidative mechanisms and the oxidative status of the blood and breast muscle of broiler chickens was investigated. In total, 240 one-day-old as hatched chicks (Ross 308) were assigned to four treatments, with four replicate pens and 15 birds in each pen. Birds were fed either a basal diet (CON) or a basal diet supplemented with 25 g/kg GGP, or 2 g/kg WYC, or 1 g starch including 100 mg pure stem extract/kg (PE) for 42 days. The polyphenolic content of vinification by-products was determined using an LC-MS/MS library indicating as prevailing compounds procyanidin B1 and B2, gallic acid, caftaric acid, (+)-catechin, quercetin, and *trans*-resveratrol. Body weight and feed consumption were not significantly affected. The relative transcript level of *GPX1* and *SOD1* tended to increase in the liver of WYC-fed broilers, while *NOX2* tended to decrease in the PE group. SOD activity in blood plasma was significantly increased in WYC and PE compared to the CON group. The total antioxidant capacity measured with FRAP assay showed significantly higher values in the breast muscle of PE-fed broilers, while the malondialdehyde concentration was significantly decreased in both WYC- and PE-fed broilers compared to the CON group. The exploitation of vinification by-products as feed additives appears to be a promising strategy to improve waste valorization and supply animals with bioactive molecules capable of improving animals’ oxidative status and products’ oxidative stability.

## 1. Introduction

Both circular economy and bioeconomy are considered as alternative economic production models that are crucial to promote sustainable growth and development [1]. The main goal of both models is to develop and achieve synergies among the economy, environment, and society. In the European Union, both agricultural and agro-industrial production processes produce annually 89 Mtons of biomass as waste [2]. Amongst these by-products, vital bioactive compounds with high added value and significant potential for utilization as feed additives are discarded as well [3,4]. Grape is considered to be the world’s largest fruit crop, with an annual production exceeding 67 Mtons. Within the Mediterranean basin area, the by-products of the winery industry are broadly available, as 62% of global wine production is located in this area [5]. Approximately 20% of the entire grapes’ biomass used for wine production is wasted. The waste is composed of stems, the woody part of the grapevine and grape pomace, and the solid residue of vinification consisting of skins, stems and seeds [6]. These biomaterials are usually discarded into nearby open fields for biodegradation, thus polluting the environment and water reservoirs in the vicinity [7]. Numerous studies have highlighted the potential of these by-products for use as plant originated feed additives that are predominantly rich in a broad variety of polyphenols [8,9], especially flavonoids, such as catechins and procyanidins with varying degrees of polymerization [10]. In this context, there are many studies and research endeavors demonstrating the powerful antioxidant properties of these polyphenols, as pure compounds and/or extracts, and their ability to act as potent free radical scavengers for the improvement of an organism’s oxidative balance [8].

Since the use of antibiotics as growth promoters was banned in the EU for poultry production in 2006, several management and nutritional strategies in the poultry industry were proposed in order to maintain high standards of productivity, healthiness, and welfare [9]. More specifically, in broilers, dietary supplementation with grape pomace appeared to effectively substitute vitamin E, preserving the antioxidant capacity in ileal content, excreta, and muscle tissues [8]. This perspective of utilizing natural compounds with vast antioxidant potentials for the substitution of synthetic substances has gained significant attention due to various safety issues raised over time [11]. On the other hand, the use of synthetic molecules such as butylated hydroxyl anisole (BHA) and butylated hydroxytoluene (BHT) has been linked with possible toxicity, which has been correlated to side effects on the liver and carcinogenesis in animal studies [11,12]. Thus, there is a growing demand of consumers towards the replacement of synthetic with natural antioxidants. The utilization of vinification by-products for the production of natural antioxidants enables producers to satisfy this trend in an economically affordable process and contributes to environment preservation affording added-value functional foods as cleaner label products.

However, the high crude fiber content in combination with some anti-nutrient factors (condensed tannins) of grape pomace may have negative nutritional effects in broilers [13]. Indeed, Kumanda et al. [14] found that the dietary inclusion of grape pomace at higher than 7.5% decreased the average feed intake in broilers. This limitation constitutes a serious drawback for the utilization of grape pomace as animal feed. Aiming to facilitate the supply of beneficial antioxidant compounds of grape pomace in animals, many strategies have been suggested, such as the treating of grape pomace with polyethylene glycol, a well-justified tannin-binding compound [13]. However, under a valorization perspective, such a process appears to be cost-ineffective and resource-consuming. The extraction of polyphenolic extracts of grape leftovers seems to be a more promising sustainable and effective strategy to supply both animals and their products with antioxidant compounds without adversely affecting growth performance and diet efficiency. Indeed, in the study of Iqbal et al. [15], the substitution of vitamin E by a polyphenolic extract of grape by-products enhanced the antioxidant status and the immunity of broilers at a lower feed cost without any side effects on the physiology or health of chickens. However, it is still questionable whether such extracts could effectively substitute the antioxidant supply of grape pomace considering both economical and physiological perspectives.

Another by-product resulting after the wine-making process with high potential as a feed additive but lacking scientific attention is wine lees. Wine lees represent approximately 5% of wine production, mainly containing ethanol, tartrate salts, phenolic compounds, and yeast cell walls [16]. According to the Greek national regulation (Joint Ministerial Decision, No. 50910/2727, 2003), ethanol that has been isolated from wine lees has received a license to be commercialized. Moreover, due to their high antioxidant capacity, wine lees have been introduced as preservatives to replace the most commonly used synthetic additives in meat products as well. Alaracon et al. [17] reported a decreased lipid and protein oxidation of deer meat that had been treated with 2.5 and 5% wine lees. Without narrowing out on wine lees’ antioxidant properties, their pivotal role as prebiotics portrays an additional important potential as feed additives as well. More specifically, a higher survival rate has been found in *Lactobacillus* and *Bifidobacterium* genera when they were treated with wine lees [18]. Although wine lees are a promising by-product for valorization, no information exists regarding their impact on animals’ performance.

Although dietary supplementation with grape by-products has been satisfactorily studied in broilers’ growth performance, scarce information exists regarding their impact on their organisms’ antioxidative status. Considering these issues, the objective of this study was to evaluate the impact of three winery by-products (grape pomace, stem extract, and wine lees) on the oxidative status of broiler chickens.

## 2. Materials and Methods

### 2.1. Experimental Procedures

#### 2.1.1. Broilers’ Trial

Two hundred forty as hatched (n = 240), 1-day-old, Aviagen Ross 308 broilers vaccinated at hatching for Marek, Infectious Bronchitis, and Newcastle Disease were obtained from a commercial hatchery. The study was conducted with respect to the guidelines of the European Union Directive on the defense of animals used for scientific purposes following directive EU 63/2010 and Council of the European Union 2010. Birds were allocated to 4 experimental treatments for 42 days. Each treatment had four floors of replicate cages of 15 broilers each. The maximum stocking density in the pens did not exceed 33 kg/m^2^ at any time, following directive 2007/43/EC. Each replicate was assigned to a clean floor cage (2 m^2^), and the birds were raised on wheat straw litter. The temperature program was set at 32 °C at week 1 and gradually reduced to 20 °C by week 6. The house environmental conditions (light and ventilation) were controlled according to commercial recommendations, and the heat was provided with a heating infrared lamp per pen.

#### 2.1.2. Solvents and Standards

All solvents used for the extractions of phenolic compounds from vinification by-products were purchased from Carlo Erba and Fisher Chemicals as analytical grade solvents. Solvents used for the LC-MS/MS determinations were obtained from J.T. Baker (water and acetonitrile) and Fisher Chemicals (formic acid) as LC–MS grade.

All standards used for the assessments of the phenolic compounds were obtained from Sigma-Aldrich (St. Louis, MO, USA), except epigallocatechin gallate and quercetin-3-*β*-D-glucoside that were provided by ExtraSynthese (Genay, France) and the acids coutaric, fertaric and caftaric, which were obtained from Phytolab (Vestenbergsgreuth, Germany), and ferulic acid bought from Fluka (Buchs, Switzerland). Folin-Ciocalteu’s reagent was purchased from Sigma-Aldrich, vanillin was obtained from Acros Organics (Geel, Belgium). Anhydrous sodium carbonate, sulfuric acid 98% and hydrochloric acid were purchased from Chem-Lab (Zedelgem, Belgium), glacial acetic acid from Sigma-Aldrich, hexahydrate aluminum chloride from Fluka (Buchs, Switzerland) and hydrate sodium acetate from Merck (Darmstadt, Germany).

### 2.2. Grape by-Product Processing and Diet Formulation

#### 2.2.1. Ground Grape Pomace

Grape pomace was sampled from the cooperative winery of Santorini Island, Greece and originated from ‘Assyriko’, a native Greek *Vitis vinifera* variety, immediately after the vinification process. It was air dried in a dark room until its humidity decreased to 13% and then ground in a hammermill (Libralon, Colle, Italy). This procedure increases the residue antioxidant compounds in grape pomace because it limits the drainage. The average composition of grape pomace is approximately 42.5% grape skins, 22.5% grape seeds, and 24.9% stems [19]. The chemical composition of grape pomace was analyzed as described by Tsiplakou et al. [20]. Grape pomace was also analyzed for fatty acids profile according to the method of O’Fallon et al. [21].

#### 2.2.2. Wine Lees Isolation and Process

Wine lees were derived after the wine-making process of a Greek *Vitis vinifera* variety, namely ‘Kotsifali’, and were kindly provided by the Laboratory of Oenology of the Agricultural University of Athens (Greece). Prior to the wine lees’ supplementation as an animal feed, a fractionation process was carried out to isolate value-added products (ethanol and tartaric acid) according to a protocol proposed by Dimou et al. [22], slightly modified. More specifically, wine lees were centrifuged (9000× *g*, 15 min, 10 °C) for the separation of the liquid from the solid phase, aiming at their further valorization. The liquid fraction was then distilled for ethanol isolation. The remaining solids were dissolved in 3.15 L deionized water per kg of dry weight and acidified with 361 mL HCl (37%, *w*/*w*) per kg dry weight. After 10 min of continuous stirring, the suspension was centrifuged (9000× *g*, 10 min, 4 °C) to separate the tartaric acid-rich solution from the solid fraction. The resulting solid precipitate, rich in yeast cells, was washed with deionized water and lyophilized. The concentration of tartaric acid and ethanol was determined with High-Performance Liquid Chromatography (HPLC) equipped with Rezex ROA-Organic acid H^+^ column and a Shimadzu RI detector as described by [7]. The crude protein content of freeze-dried wine lees was determined according to the Association of Official Analytical Chemists (1984) using a Kjeldahl Distillation System (FOSS Kjeltec 8400, Hilleroed, Demark).

#### 2.2.3. Extraction of Polyphenols by Grape Stems

Grape stems from native *Vitis vinifera* variety ‘Assyrtiko’, grown in the island of Santorini, were dried naturally in a dark room until their humidity reached 13% and then ground in a hammermill (Libralon, Colle, Italy) with a 1-mm sieve. The grape stem powder was consequently extracted in an Ultra Sonic bath (DiaSonic Extractor Mod. 20 L) for 15 min (38 KHz–350 Watts) in methanol-water solution (7/3). The extract was filtered and the solvent was removed using rotary evaporation (Comecta SA Rotary Evaporator R coupled with Eyela Cool Ace CA-1111) to provide a semisolid residue, which was finally dried to an amorphous solid over a freeze-dryer (18N).

**Figure 1:**
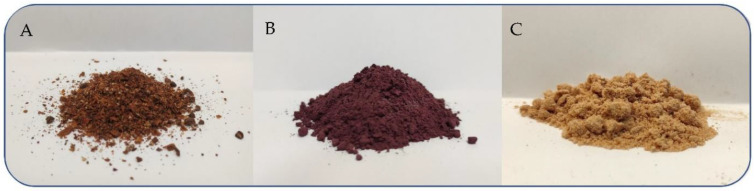
Feed additives supplemented in broiler diets: (**A**) ground grape pomace (GGP), (**B**) dried wine lees extract (rich in yeast cell walls; WYC), and (**C**) extract from grape stems included in soluble starch (PE).

This dried polyphenol extract was used to prepare the administration formula. For this purpose, 40 g of polyphenol extract was diluted in 400 mL of water and then 400 g of starch (Starch soluble GR for analysis ISO. CAS 9005-84-9). The solution was homogenized in an ultrasonic bath and then subjected to deep freezing (−80 °C). Finally, the frozen solution was dehydrated in a freeze dryer, concluding the administration formulation (Figure 1).

#### 2.2.4. Diets’ Formulation

Broilers were fed three different diets depending on growing phase: starter (0–10 day), grower (11–24 day), and finisher (25–42 day). In the control (CON) group, broilers were fed a basal diet based on corn and soybean meal. In the GGP group, ground grape pomace was added to the starter, grower, and finisher diet at a level of 2.5% (25 g/kg feed; Table 1). The inclusion rate was selected in order to achieve similar dietary metabolizable energy and crude protein content (Ross 308 Broiler Nutrition Specifications, Aviagen 2019), while the high content of crude fiber of grape pomace appeared to be the limiting factor for a higher inclusion level. In the WYC group, dried wine lees (yeast cell walls) were added to the starter, grower, and finisher diet at a level of 0.2% (2 g/kg feed; Table 1). Commercialized yeast-based products are included in broiler chickens diets up to 1 g/kg feed (1.0 × 10^6^ or 1.0 × 10^7^ CFU) [23]. The inclusion level of the present study was set based on the purity of wine lees in yeast cell content through their protein levels. In the PE group, an extract derived from grape stems using soluble starch for its inclusion (10% pure phenolic extract) was added to the starter, grower, and finisher diet at a level of 0.1% (1 g/kg feed; Table 1). The inclusion level of stems’ polyphenolic extract was set after extensively reviewing the effect of different levels and polyphenolic extract on the antioxidative status of poultry [24,25].

The diet composition is presented in Table 1. Feed and water were provided ad libitum. Experimental diets from the three growing phases were milled through a 1-mm screen before analysis. Diets’ chemical composition was performed as described above [19], and determined and calculated analyses are presented in Table 2.

### 2.3. Determination of Grape by-Products’ Antioxidants Compounds

#### 2.3.1. Extraction of Polyphenolic Compounds for the Analysis

The pomace and wine lees sample extractions were performed using a modification of the protocol developed by Anastasiadi et al. [26]. Briefly, 50 g of dried, powdered sample was extracted with 200 mL of methanolic mixture (MeOH/H_2_O/1.0 N HCl (90:9.5:0.5 *v*/*v*)) and sonicated in an ultrasonic bath (35 kHz) for 10 min. The solvent was separated by filtration, and the residual solid was extracted two additional times under the same conditions. The extracts were combined, and their solvents were evaporated under vacuum to result in a slurry, which was dissolved in 30 mL of MeOH/H_2_O (1:1) and centrifuged for 10 min at 7500 rpm. The supernatant liquid was extracted using petroleum ether (3 × 30 mL) for the extraction of lipids and the combined extracts were concentrated under vacuum. The residue was poured into 30 mL of brine and extracted repetitively with ethyl acetate (EtOAc, 4 × 30 mL) to remove sugars into the aqueous layer. The combined organic layers were dried over anhydrous MgSO_4_ and evaporated under vacuum. The remaining solid was weighed and dissolved in methanol (MeOH) to 1 mg/mL and subjected to LC-MS/MS analysis. To avoid polyphenol degradation, all above-mentioned activities were performed in the absence of direct sunlight and at temperatures below 35 °C. The estimation of polyphenolic profile of grape stems was performed on the extract used for the preparation of the feed additive.

#### 2.3.2. Estimation of Polyphenolic Compound Presence

The estimation of polyphenolic profiles in vinification by-products was performed by the spectrophotometric evaluation of their Total Phenolic Content (TPC), Total Flavonoid Content (TFC) and Total Tannin Content (TTC). The respective data were recorded on an Infinite^®^ 200 PRO instrument (Tecan Group Ltd., San Jose, CA, USA).

##### Determination of Total Phenolic Content (TPC)

The TPC was measured by the spectrophotometric method of Hilma et al. [27] with some modifications. In particular, 10 μL of each sample was placed in a 96-well plate (Sarstedt AG & Co. KG, Nümbrecht, Germany) and 100 μL of water and 10 μL of Folin-Ciocalteu reagent solution were added. After 3 min of incubation at room temperature, 20 µL of Na_2_CO_3_ in aqueous solution (7.5% *w*/*v*) and an additional 60 μL of water were added. The solution was incubated in the dark for 60 min and the absorbance was determined at 765 nm. The quantification of each sample was based on its comparison against a standard curve created in a range of 30–200 µg/mL (30, 55, 80, 110, 135, 160, 180, 200 µg/mL) solutions of gallic acid in methanol. Results are expressed as gallic acid equivalents in dry weight (DW) of each sample.

##### Determination of Total Flavonoid Content (TFC)

The TFC was determined by modifying the aluminum chloride method of Pękal and Pyrzynska [28]. Specifically, 100 µL of sample was mixed with 50 µL of AlCl_3_ aqueous solution (2% *w*/*v*) and 50 µL of CH_3_COONa in water (1 M) and placed in a 96-well plate. The mixture was incubated in the dark at room temperature for 40 min and the absorbance was measured at 415 nm in a microplate reader. The TFC value of each sample was calculated against a standard calibration curve of quercetin in methanol with concentrations of 10, 25, 40, 55, 70, 85, 100 µg/mL. Results are expressed as quercetin equivalents (QE) in dry weight (DW) of each sample.

##### Determination of Total Tannin Content (TTC)

The TTC was estimated by a modified version of the method developed by Hong et al. [29]. Briefly, 25 µL of sample was mixed with 150 µL of vanillin methanolic solution (4% *w*/*v*) in a 96-well plate and 25 µL 32% H_2_SO_4_ in methanol was added. The mixture was incubated for 15 min at 25 °C and the absorbance was measured at 500 nm in a microplate reader. The results were obtained using a standard calibration curve of epicatechin solution in methanol at concentrations of 120, 220, 350 500, 650, 800, 950, 1000 µg/mL. Results are expressed as g of epicatechin (EE) equivalents in dry weight (DW) of each sample.

#### 2.3.3. Identification and Quantification of Polyphenolic Compounds by LC-MS/MS Analysis

##### Analytical Solutions and Sample Preparation

Stock solutions of each analyte were prepared in methanol for concentrations ranging from 90 to 2400 μg/mL. The stock solutions were maintained at −20 °C and used for the preparation of an intermediate methanolic stock solution containing all analytes for 20 μg/mL concentration. Before each analysis, the respective stock solutions were diluted in concentrations ranging from 50 to 1500 ng/mL. The latter were utilized for the construction of calibration curves immediately prior to sample analyses. The samples of the extracts were prepared by diluting 1 g of extract in 1 mL of methanol just before the analysis. All standards solutions and all the samples were analyzed in triplicate.

##### LC-MS/MS Analysis

LC-MS/MS was selected as the analytical method for assessment of phenolic compound presence because of its selectivity and sensitivity [30]. The identification of phenolic compounds was performed using an Accela Ultra-High-Performance Liquid Chromatography system coupled with a TSQ Quantum Access triple quadrupole mass spectrometer equipped with an autosampler (Thermo Fischer Scientific, Waltham, MA, USA).

The stationary phase of the chromatographic analysis was a C18 column (Fortis Technologies Ltd. Neston, UK; C18, 150 × 2.1 mm, 3 μm) with a guard column (10 × 2 mm, 3 μm) of the same material and company. The mobile phase consisted of two solutions, both containing formic acid (0.1%) and water (A) or acetonitrile (B). The mobile phase gradient program was: 0.0–2.0 min: 10% B, 2.0–16.7 min from 10% B to 100%, 16.7–18.7 min 100% B, and 18.8–22.0 min 10% B to re-equilibrate the column. The flow rate was 0.2 mL/min. The injection volume was 10 μL and the temperature of the tray and the column was set at 25 and 35 °C, respectively.

Mass spectrometer was operated on electrospray ionization (ESI) technique in negative and positive polarities and the selected reaction monitoring (SRM) mode for increased sensitivity. Before each analysis, all target analytes’ molecular ion transitions and their collision energies were obtained by direct infusion in full scan (mass range: 100–1500). The ion source and vacuum parameters were optimized to be applicable for all analytes. A nitrogen generator (Peak Scientific) was used to generate nitrogen as sheath and auxiliary gas. The respective gas pressures were set at 25 and 10 Arb, respectively. The spray voltage was set at 3.5 kV in the negative polarity and 3.0 kV in the positive polarity, capillary temperature was regulated at 300 °C, and collision pressure was adjusted at 1.5 mTorr.

The signals of the selected ion transitions of the deprotonated molecules of *m*/*z* used were: gallic acid (169.939 > 126.089 (17 eV), 169.939 > 125.047 (17 eV)), caftaric acid (312.151 > 149.039 (14 eV), 312.151 > 179.985 (17 eV)), procyanidin B1 (578.328 > 426.099 (18 eV)), epigallocatechin (306.138 > 124.855 (27 eV), 179.658 (18 eV)), chlorogenic acid (854.200 > 124.855 (27 eV), 354.200 > 191.113 (20 eV)), catechin (290.133 > 203.958 (22 eV), 290.133 > 245.958 (17 eV)), procyanidin B2 (578.122 > 290.047 (31 eV)), coutaric acid (296.129 > 120.145 (29 eV), 296.129 > 164.015 (18 eV)), fertaric acid (326.172 > 134.113 (33 eV), 326.172 > 194.059 (18 e V)), epicatechin (290.132 > 203.818 (21 eV), 290.132 > 245.948 (17 eV)), epigallocatechin gallate (458.233 > 167.890 (22 eV), 458.233 > 457.460 (11 eV)), caffeic acid (180.102 > 135.095 (24 eV), 180.102 > 136.106 (19 eV)), syringic acid (198.085 > 167.890 (22 eV), 198.085 > 182.921 (22 eV)), polydatin (390.548 > 229.992 (21 eV), 390.548 > 389.777 (8 eV)), quercetin-3-b-D-glucoside (464.220 > 300.781 (28 eV), 464.220 > 301.966 (26 eV)), epicatechin gallate (442.252 > 168.845 (23 eV), 442.252 > 290.236 (21 eV)), procyanidin A2 (576.358 > 424.052 (18 eV), 576.358 > 449.815 (24 eV)), rutin (610.355 > 271.536 (68 eV), 610.355 > 302.205 (44 eV)), p-coumaric acid (164.014 > 94.475 (36 eV), 164.014 > 119.835 (18 eV)), sinapic acid (224.132 > 193.987 (24 eV), 224.132 > 209.043 (17 eV)), ferulic acid (194.120 > 135.094 (20 eV), 194.20 > 179.062 (16 eV)), myricetin (318.114 > 136.790 (29 eV), 318.114 > 178.963 (22 eV)), o-coumaric acid (163.970 > 119.068 (17 eV), 163.970 > 11120.127(16 eV)), trans-resveratrol (228.146 > 144.131 (29 eV), 228.146 > 186.109 (22 eV)), quercetin (302.111 > 151.483 (24 eV), 302.111 > 179.692 (22 eV)), apigenin (270.037 > 116.922 (42 eV), 270.037 > 117.972 (40 eV)), kaempferol (286.102 > 211.942 (33 eV), 286.102 > 229.944 (27 eV)), isorhamnetin (316.376 > 301.277 (24 eV), 316.376 > 302.404 (23 eV)), internal standard (2-(4-chlorophenyl)malonaldehyde) (182.456 > 136.900 (26 eV), 182.456 > 154.892 (19 eV)). Oenin (493.236 > 315.121 (47 eV), 493.236 > 331.122 (23 eV)) was the only analyte detected as a protonated molecule.

### 2.4. Determination of Performance Parameters

Body weight (BW) was recorded on the first day of the experimental period and at the end of each feeding phase. Feed intake was recorded and feed conversion ratio (FCR) was calculated. Total mortality was calculated as the number of broilers that died throughout the study compared to the initial number of broilers placed.

### 2.5. Sample Collection

At the age of 42 days, 32 broilers (8 per treatment and 2 per replicate pen) were randomly selected and sacrificed. Blood was collected while the liver tissue was carefully excised and immediately snap-frozen and subsequently stored at −80 °C for further analyses. Approximately 6 mL of whole blood was immediately transferred to heparin-containing tubes (170 units heparin; BD Vacutainer, Plymouth, UK) and stored in an icebox (Thomas Scientific, Swedesboro, NJ, USA) until its transfer to the Laboratory of Nutritional Physiology and Feeding. Then, the blood samples were centrifuged (SL16R, Thermo Fisher Scientific, Waltham, MA, USA) at 2500 rpm for 15 min at 4 °C to separate plasma from the cells. Additionally, the breast muscle was also collected.

### 2.6. Molecular Analysis

#### 2.6.1. RNA Isolation and cDNA Synthesis

Total RNA was isolated from the liver tissue samples of broilers separately using Trizol (Invitrogen, CA, USA) according to the manufacturer’s instructions. The quantity and quality of the extracted RNA were confirmed by spectrophotometry (NanoDrop ND-1000) via ng/μL and purity was determined by the ratios A260/A280 and A260/A230; in addition, agarose gel electrophoresis was performed for each sample to examine any degradation. Approximately 5 μg of each extracted RNA was treated with Turbo DNAse (2 Units/μL) using a commercially available kit (Invitrogen, CA, USA). After DNase treatment, RNA was correlated with the positive control (*Gallus gallus* genomic DNA) as a template, using glyceraldehyde 3-phosphate dehydrogenase (*GAPDH*) as housekeeping gene and a Taq polymerase PCR protocol to investigate, in agarose gel, the absence of DNA contamination. Then, RNA was further purified with a phenol:chloroform protocol, and pure RNA was precipitated. The quantity and quality of the pure RNA were confirmed again by spectrophotometry (NanoDrop ND-1000) as well as by agarose gel visualizing the 28S and 18S ribosomal RNA. Approximately 70% yield of RNA was recovered after DNase treatment. Pure RNA (500 ng) was reverse-transcribed with the PrimeScript First Strand cDNA Synthesis Kit (Takara, Shiga, Japan), according to the manufacturer’s instructions using a mix of random hexamers and oligo-dT primers.

#### 2.6.2. Primers’ Design

A pair of primers specific for *GAPDH*, Glutathione Peroxidase 1 (*GPX1*), Glutathione Peroxidase 2 (*GPX2*), NADPH oxidase 1 (*NOX1*), NADPH oxidase 2 (*NOX2*), and NADPH oxidase 3 (*NOX3*) genes were designed using Geneious software (Biomatters Ltd., Auckland, New Zealand) according to the respective *Gallus gallus* gene coding sequences (CDS in GenBank) (Table 3). Additionally, a set of primers specific for Catalase (*CAT*), Superoxide Dismutase 1 (*SOD1*), Glutathione Transferase A2 (*GSTA2*), Nitic Oxide Synthase 2 (*NOS2*), and Beta-actin (*ACTB*), which have been previously initiated by Ahmadipour et al. [31], Ibrahim et al. [32], Ebrahimi et al. [33], and Paraskeuas and Mountzouris [34], were used. The specificity of each pair of primers was tested through the dissociation curves, and the amplification products were subjected to agarose gel electrophoresis to confirm the production of a single amplicon per reaction.

#### 2.6.3. Real-Time Quantitative PCR

The relative mRNA expression levels for the target genes were quantified with a StepOnePlusTM Real-Time PCR System (Applied Biosystems, Foster City, CA, USA) as described by Mavrommatis et al. [35]. *GAPDH* and *ACTB* were used as housekeeping genes to normalize the cDNA template concentrations [36]. The relative expression levels of the target genes were performed as described by Mavrommatis et al. [35], while the primers’ efficiency was calculated by employing the linear regression method on the log (fluorescence) per cycle number (ΔRn) using the LinRegPCR software [37].

### 2.7. Biochemical Analyses

#### 2.7.1. Antioxidant Enzyme Activities and Oxidative Status Indicators in Blood Plasma

The assays for antioxidant enzyme activities, oxidative stress indicators, and the total antioxidant capacity were performed using a UV/Vis spectrophotometer (GENESYS 180, Thermo Fisher Scientific, Waltham, MA, USA) as previously described [38]. The GST activities were recorded by monitoring the conjunction of GSH to 1-chloro-2,4-dinitrobenzene (CDNT) at 340 nm. CAT activity was performed using a commercial spectrophotometric kit (Catalase Assay Kit; CAT100, Sigma-Aldrich, St. Louis, MO, USA). GSH-Px activity was assayed according to Paglia and Valentine [39]. GR activity was performed by measuring the reduction in oxidized glutathione (GSSG) to reduce glutathione in the presence of nicotinamide adenine dinucleotide phosphate (NADPH) at 340 nm. SOD activity was recorded by monitoring the inhibition of cytochrome c oxidation at 550 nm. LPO activity was performed by monitoring the oxidation of 2,2′-Azino-bis (3-ethylbenzthiazoline-6-sulfonic acid) ABTS in the presence of hydrogen peroxide at 340 nm. MDA was measured according to Nielsen et al. [40] with some modifications described by Mavrommatis et al. [35]. The protein carbonyls (PC) were assayed according to the method of Patsoukis et al. [41]. The ABTS [42,43] and the ferric reducing ability of plasma (FRAP) [44] assays were used to assess the total antioxidant capacity.

#### 2.7.2. Breast Muscle Antioxidant Status

Lipid peroxidation activity in breast muscles: the sample extraction carried out using the method of Park et al. [45]. Briefly, 2 g of breast muscle sample was homogenized with 6 mL of distilled water using a homogenizer (THP115, Omni TH, Kennesaw, GA, USA). After that, the homogenate was used for the determination of the MDA-TBA complex. Lipid peroxidation was assayed by measuring malondialdehyde (MDA) according to the method of Nielsen et al. [40].

Determination of antioxidant activity by the FRAP, ABTS, and DPPH assays: the sample extraction was carried out using a modified method of Martínez et al. [46]. More specifically, 2 g of breast muscle sample was homogenized with 18.5 mL of 25% ethanol (*v*/*v*) using a homogenizer and was shaken for 1 h at 680 rpm at room temperature in a rotary shaker (ZWY-304, Labwit Scientific Pty Ltd., Victoria, Australia). The mixture was then centrifuged for 4 min at 3500× *g* and 4 °C. The supernatant was filtered through Whatman filter paper. The filtered samples were used for FRAP, ABTS, and DPPH methods. Ferric reducing antioxidant power (FRAP) assay was used to measure total antioxidant potential according to Benzie and Strain [44]. ABTS radical scavenging capacity assay was based on the published methods [42,43], while the measurement of the scavenging capacity was estimated by the DPPH method [47].

### 2.8. Statistics

Dataset was evaluated in SPSS.IBM software (v 20.0) and the results are depicted as mean ± standard error of means (SEM). For broilers’ growth performance, each experimental unit consisted of the replicate pen, while for molecular and biochemical analyses the experimental unit considered the animal. Dietary treatment effects were explored using one-way analysis of variance (ANOVA) followed by Fisher’s Least Significant Difference multiple range test. Simplifying the visualization of these results, GraphPad Prism 6.0 (2012) depicted interleaved bars ± SEM. Statistical significance was set at *p* ≤ 0.05. Sex was not included in the statistical model because, at the farm level, the broilers’ sexing is not practiced.

Discriminant analyses were also performed (variables were entered independently together) on liver relative expression levels of selected genes and the antioxidant indicators of both blood plasma and breast muscles to establish those variables capable of distinguishing and classifying samples amongst the four dietary groups (CON, GGP, WYC, and PE). Wilks’ lambda (λ) criterion was used for assessing discriminant functions [48]. Twenty-two variables were entered to create a model to distinguish the thirty-two samples of each case (4 dietary groups × 4 pen replicates/dietary group × 2 animal/pen). Principal component analysis (PCA) was applied to the pooled data of liver relative expression levels of selected genes and the antioxidant indicators of both blood plasma and breast muscles in order to reduce the dimensionality of the data and to underline the relationships between the variables. Twenty-two variables from thirty-two samples each were entered into a model to investigate their communalities (extraction > 0.6). Parallel analysis was also applied to eigenvalue plot to find out the number of factors to extract, while the Kaiser-Meyer-Olkin (KMO) criterion was applied to assess PCA dependability. The contribution of variables on its component was examined by suppressing small coefficients to >0.3.

## 3. Results

### 3.1. Grape by-Products’ Composition and Polyphenol Profile

Table 4 presents the chemical composition and the fatty acid profile of ground grape pomace supplemented in the GGP group. Crude protein was measured at 8% while grape pomace was determined as rich in crude fiber (24.27%). More specifically, the acid detergent fiber (ADF) was recorded at 34.65% and the neutral detergent fiber (NDF) at 37.10%. Stearic (4%), oleic (19.33%), and linoleic (62.42%) acid were found as the prevailing fatty acids in grape pomaces (Table 4).

Wine lees obtained from the ‘Kotsifali’ wine production consisted of 26.8 g solids per 100 g of wine lees, while the remaining 73.2 g of liquid contained 12.4% (*w*/*w*) ethanol. The acidification of the solid fraction resulted in 7.18 g tartaric acid per 100 g of dry lees (10.41 g/L). Tartaric acid could effectively be used as a food additive, regulating the acidity of the final product. The crude protein content of wine lees was assayed, aiming to indirectly estimate yeast cell content. The solid fraction remaining after tartaric acid isolation contained 25% protein, reflecting approximately 62.5% yeast cells based on [16].

The Total Phenolic Content, Total Flavonoid Content, and Total Tannin Content of grape by-products used in feed formulation are included in Table 5. As shown, the respective values of TPC, TFC, and TTC for pomace (GGP) were significantly higher as compared to those determined for stems (PE) and wine lees (WYC). It must be noted that the measurements reflect the quantities included in the corresponding dietary treatments.

The results of the quantization of phenolic compounds content in samples, determined by LC-MS/MS analysis, are included in Table 6. The method was optimized, and the limit of detection (LOD) and limit of quantification (LOQ) values ranged from 7.5 to 158.3 ng/mL and 22.6 to 479.8 ng/mL, respectively.

### 3.2. Growth Performance

In Table 7, the effects of feeding ground grape pomace (GGP), dried wine lees extract (WYC), and grape stem extract (PE) on broilers’ body weight, feed consumption, and FCR are presented. Considering the whole experimental period (42 days), the growth performance was not considerably affected. Nevertheless, the final BW of the PE group was numerically increased by 4%. Mortality rate tended to increase (*p* = 0.079) in the WYC group compared to CON.

### 3.3. Relative Transcript Levels of Genes Involved in Oxidative Status in Liver

**Figure 2:**
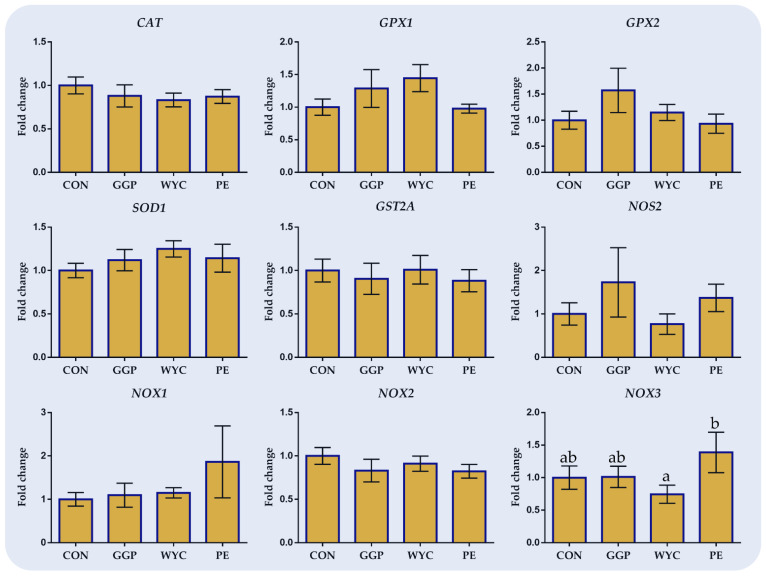
Mean and standard error of means (SEM) of relative transcript levels as fold changes of several genes involved in the antioxidant system in the liver of broilers fed the four experimental diets (Control; CON, ground grape pomace; GGP, dried wine lees; WYC, and extract from grape stems included in soluble starch; PE). Bars with different superscript (a, b) between dietary treatments differ significantly (*p* ≤ 0.05) according to the analysis of variance (ANOVA) using post hoc multiple range test when appropriate.

In Figure 2, the effects of feeding ground grape pomace (GGP), wine lees (WYC), and grape stem extract (PE) on the relative expression of genes involved in oxidative mechanisms in the liver of broilers are presented. Although no significant (*p* > 0.05) alterations were observed in the relative expression of the investigated genes between CON and the dietary treatments (GGP, WYC, and PE), few tendencies were revealed. More specifically, the relative transcript levels of *GPX1* and *SOD1* tended to increase in the WYC group by 40% (*p* = 0.092) and 30% (*p* = 0.093), respectively (Figure 2). *NOS2* showed an upward trend in the liver of GGP- and PE-compared to CON-fed broilers without the results being significant (Figure 2). Similarly, *NOX1* and *NOX3* relative transcript levels were numerically increased in the PE compared to the CON group, while in the PE- compared to WYC-fed broilers a significant upregulation (*p* = 0.039) of *NOX3* was found. *NOX2* tended to decrease (*p* = 0.098) in the liver of PE-fed broilers compared to the CON group (Figure 2).

### 3.4. Antioxidant Enzyme Activities, Total Antioxidant Capacity, and Oxidative Status in Blood Plasma

**Figure 3:**
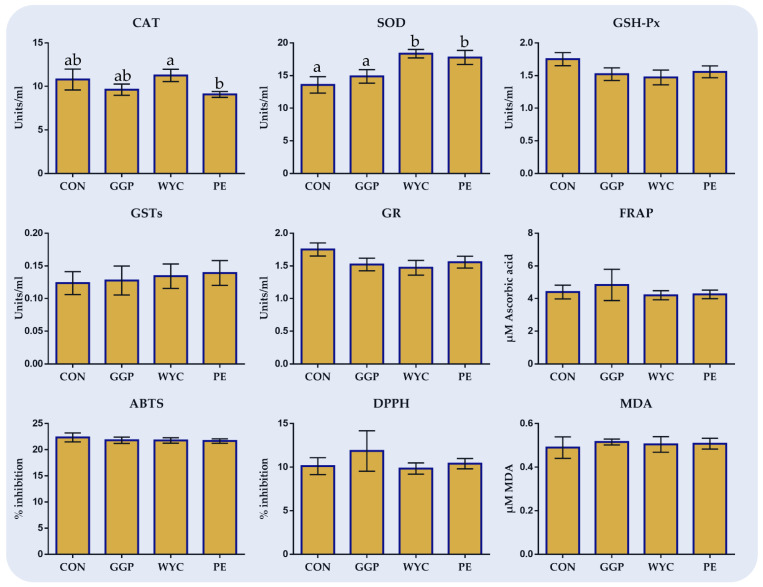
Means ± SEM of total antioxidant capacity, oxidative stress indicators, and enzyme activities (Units/mL) in the blood plasma of broilers fed the four diets (Control; CON, ground grape pomace; GGP, dried wine lees extract; WYC, and grape stem extract included in soluble starch; PE) at 42 days. Bars with different superscript (a, b) between dietary treatments differ significantly (*p* ≤ 0.05) according to the analysis of variance (ANOVA) using post hoc multiple range test when appropriate.

In Figure 3, the effects of feeding ground grape pomace (GGP), wine lees extract (WYC), and grape stem extract (PE) on the antioxidant enzyme activities, total antioxidant capacity, and oxidative status in the blood plasma of broilers are presented. Catalase activity was significantly increased (*p* = 0.049) in WYC compared to PE-fed broilers. On the other hand, SOD activity was significantly increased in WYC (*p* = 0.003) and PE (*p* = 0.007) compared to the CON group (Figure 3). The activity of GSH-Px in the blood plasma of WYC-fed broilers tended to decrease (*p* = 0.060) compared to the CON group (Figure 3). The total antioxidant capacity of blood plasma estimated by FRAP, ABST, and DPPH methods and the lipid peroxidation indicator (MDA) did not differ amongst the dietary groups.

### 3.5. Breast Muscle Total Antioxidant Capacity and Lipid Peroxidation Indices

**Figure 4:**
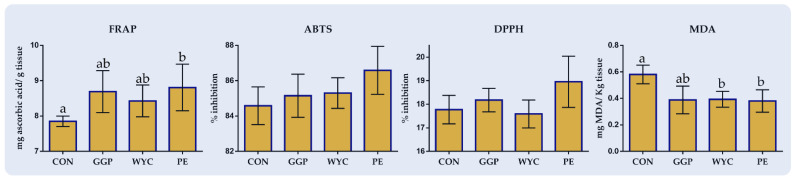
Total antioxidant capacity and lipid peroxidation index of breast muscle of broilers fed the four diets (Control; CON, ground grape pomace; GGP, dried wine lees extract; WYC, and grape stem extract included in soluble starch; PE) at 42 days. Bars with different superscript (a, b) between dietary treatments differ significantly (*p* ≤ 0.05) according to the analysis of variance (ANOVA) using post hoc multiple range test when appropriate.

In Figure 4, the effects of feeding ground grape pomace (GGP), wine lees extract (WYC), and grape stem extract (PE) on the total antioxidant capacity and lipid peroxidation indicator of the breast muscle of broilers are depicted. The total antioxidant capacity measured by FRAP assay showed a significantly higher value (*p* = 0.044) in the breast muscle of PE-fed broilers compared to the CON group (Figure 3). The MDA concentration of breast muscle at 48 h at 4 °C was significantly decreased in both WYC-(*p* = 0.047) and PE-fed (*p* = 0.042) broilers (Figure 4).

**Figure 5:**
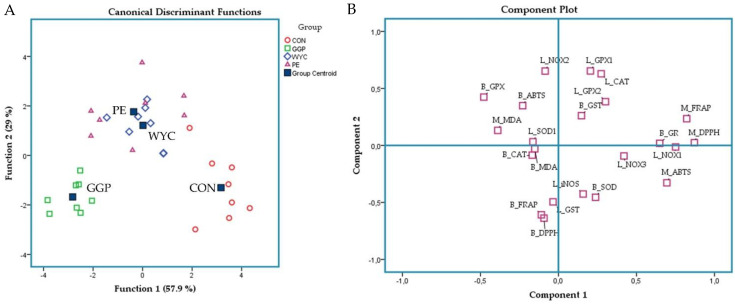
(**A**) Discriminant plots separating the four dietary treatments (Control; CON red, ground grape pomace; GGP green, dried winery yeast cell walls; WYC blue, and phenolic extract from grape stems included in soluble starch; PE purple) according to pooled data of the liver’s relative transcript levels and blood plasma and breast muscle antioxidant indicators. (**B**) Principal component analyses were applied on liver relative transcript levels, blood plasma, and breast muscle antioxidant indicators. L_CAT: catalase relative expression in liver; L_SOD1: superoxide dismutase 1 relative expression in liver; L_GPX1: glutathione peroxidase 1 relative expression in liver; L_GPX2: glutathione peroxidase 2 relative expression in liver; L_iNOS: nitrous oxide relative expression in liver; L_NOX1: NADPH oxidase 1 relative expression in liver; L_NOX2: NADPH oxidase 2 relative expression in liver; L_NOX3: NADPH oxidase 3 relative expression in liver; L_GST: glutathione transferase 2A relative expression in liver; B_FRAP: blood plasma FRAP value; B_ABTS: blood plasma ABTS value; B_DPPH: blood plasma DPPH value; B_MDA: blood plasma MDA concentration; B_CAT: catalase activity in blood plasma; B_SOD: superoxide dismutase activity in blood plasma; B_GPX: glutathione peroxidase activity in blood plasma; B_GST: glutathione transferase activity in blood plasma; B_GR: glutathione reductase activity in blood plasma; M_FRAP: FRAP value in breast muscle; M_ABTS: ABTS value in breast muscle; M_DPPH: DPPH value in breast muscle; M_MDA: MDA concentration in breast muscle.

A discriminant analysis was also applied to the pooled data of liver relative transcript levels, blood plasma, and breast muscle antioxidant indicators to establish those variables capable of distinguishing and classifying samples among the four dietary groups (Figure 5A). The proportions of the samples that were correctly classified were 93.8%. Wilks’ (λ) was reported at 0.021 for Function 1 (*p* = 0.352) and 0.128 for Function 2 (*p* = 0691), while the SOD activity in blood plasma was the variable that contributed the most. Although the Wilks’ (λ) values supported clear discrimination, the limited dataset underperformed its significance. Nevertheless, the CON group has been allocated into the right-down corner apart from grape-supplemented groups. WYC and PE groups showed a major overlap, indicating a comparable response. Principal component analysis was also performed to reduce the dimensionality of the variables (Figure 5B). Total antioxidant capacity measured by FRAP, ABTS, and DPPH assays between blood plasma and breast muscle was oppositely allocated, indicating a negative correlation (Figure 5B). Blood plasma and breast muscle MDA content showed a negative correlation with breast muscle total antioxidant capacity (Figure 5B). The relative transcript levels of NOX1 and NOX3 were also oppositely placed from blood plasma GSH-Px activity as well (Figure 5B).

## 4. Discussion

Many studies have underlined the importance of vinification by-products as plant materials particularly rich in a broad variety of polyphenols. Grape seeds and skins contained in grape pomace constitute important sources of flavonoids, mainly catechins and procyanidins [6,7]. The implementation of LC-MS/MS analysis proved to be a tool of implacable importance that provided us a broad screening of the polyphenolic composition present in grape by-products. Amongst them, procyanidin B1 and B2, gallic acid, caftaric acid, catechin, quercetin, and resveratrol were determined as the prevailing compounds. In a study concerning the analysis of grape seeds from both red and white varieties, they were also found to be rich in catechin, epicatechin, epicatechin, protocatechic acid, procyanidin B1, procyanidin B2, procyanidin B3, and procyanidin B4. Lower concentrations of gallic acid and protocatechic acid were found as well [49]. Puiggròs et al. [50] demonstrated that grape seed procyanidin extracts are capable of modulating the expression of antioxidant systems, indicating that procyanidin extracts of grape seeds could act to improve the cellular oxidative status through glutathione synthesis pathways. Additionally, Preuss et al. [51] have reported that the supplementation of proanthocyanidin extracts from grape seeds significantly decreased the concentrations of total cholesterol, oxidized LDL, and LDL after two months in hypercholesterolemic participants.

Numerous enzymes and cellular processes produce reactive oxygen species (ROS), including the mitochondrial electron transport chain, nitric oxide synthases (NOSs), cytochrome P450 reductase, and xanthine oxidase. Nevertheless, in most of these mechanisms, ROS formation results as a by-product of their catalytic function or from a dysfunctional variant of the enzyme. On the contrary, NADPH oxidases are the only enzymes whose principal role is to generate superoxide and consequently other ROS [52]. NOX family proteins are the catalytic, electron-transporting subunits of the NADPH oxidase enzyme complex [53]. In our study, the relative transcript level of NOX2 tended to decrease in the liver of PE-fed birds, indicating a lower production of ROS. Interestingly, it has been found that polyphenols, excepting their well-documented ROS scavenging abilities, downregulate NADPH oxidase in numerous tissues of rats and humans including, but not limited to, vessels and platelets [54,55]. Notably, new entry polyphenolic compounds that lack typical superoxide scavenging properties and directly inhibit NOX activity are being studied. Steffen et al. [56] tested the role of several polyphenols in oxidative stress by establishing a set of structural requirements for scavenging ROS and inhibiting NADPH oxidase function. More specifically, polyphenols such as catechin, epicatechin, quercetin, luteolin, and fisetin scavenge the unstable superoxide due to their lack of additional substitutions in their B ring. However, the intake of the aforementioned compounds was not higher in the PE groups. These sets of evidence support that the polyphenols’ composition (profile) may tightly regulate the in vivo antioxidant mechanisms rather than their absolute level per se.

The inclusion of dried wine lees extracts in broilers’ diets tended to increase the relative expression levels of *SOD1* and *GPX1* in the liver of WYC-fed birds. The study of Spanier et al. [57] showed that resveratrol, an effective polyphenol contained in grapes, reduced NOX4 while increasing SOD1 and GPX1 mRNA levels in human umbilical endothelial cells, which was associated with reduced ROS levels. Furthermore, Hu et al. [58] reported that dietary supplementation of resveratrol can inhibit lipid peroxidation and improve antioxidant enzymes’ (SOD, GSH-Px, CAT) activity in rats’ hepatocytes as well. Interestingly, the intake of resveratrol in WYC groups was lower compared to other grape by-product treatments, showing the potential involvement of other compounds in SOD1 and GPX1 regulation. The rise of SOD mRNA levels in broilers’ liver caused an increase in the enzymatic level in the blood of WYC- and PE-fed groups compared to the CON. SODs depict the first defense against ROS-mediated injury. SODs catalyze the dismutation of superoxide anion free radical (O_2_^−^) into molecular oxygen and the less harmful ROS, hydrogen peroxide (H_2_O_2_), decreasing the O_2_^−^ level, which damages the cells at extensive concentrations [59]. In compliance with our findings, supplementation with *Echinacea purpurea* L. rich in caftaric and cichoric acids in broilers’ diets increased the activity of SOD in both blood serum and liver [60].

On the contrary, GSH-Px activity in blood tended to decrease in the WYC group even though the GPX1 mRNA levels in the liver tended to increase. Overlooking the potential involvement of post-transcriptional factors and discrepancies amongst tissue responses [61], the suppression of GSH-Px activity in blood may lie in SOD upregulation. More specifically, the neutralization of superoxide anion by SOD in blood and subsequently the formation of hydrogen peroxide, the main substrate of both CAT and GSH-Px, may result in an inhibitory feedback. Still, it has been previously found that a high concentration of H_2_O_2_ inhibits the activity of GSH-Px [62], while the hydroxyl radical formed by a Fenton reaction using H_2_O_2_ as a substrate inhibits CAT activity as well [63].

Total antioxidant capacity was measured in both blood plasma and breast muscles, aiming to investigate the overall oxidative status of tissues. Three methods (FRAP, ABTS, and DPPH) were used, aiming to expand methods’ completability by observing a much broader and dependable perspective. Notably, the FRAP method underestimates the level of the principal exogenous antioxidant, glutathione, compared to the ABTS method [64]. Concerning the FRAP method, its principal contributors in mammals’ biological samples are uric acid, α-tocopherol, bilirubin, and ascorbic acid, while it does not evaluate the SH-group encompassing non-enzymatic antioxidants, such as glutathione and albumin [64]. DPPH is a rarely used method in biological samples since the organic compounds are precipitated in their alcoholic medium [64]. However, DPPH rather complements other methods since it measures substances of non-protein origin substances. Therefore, since there is no optimal assay to evaluate the total antioxidant potential of biological samples, the synergistic use of various assays is strongly suggested, validating changes in the total antioxidant status. In our study, although TAC did not differ in blood plasma, a significant increase in the breast muscle of PE-fed broilers was found according to the FRAP method. This result may be correlated to the overall improvement in the oxidative status in PE groups resulting from *NOX2* downregulation in the liver and an SOD increase in blood plasma.

MDA is one of the main intermediates between lipid peroxidation and oxidative stress [40]. The rise in MDA levels, which reflects the degree of lipid peroxidation, could be attributed to the increased levels of ROS. Even though blood plasma MDA levels were not affected amongst the dietary treatments, their levels in the breast muscle of WYC- and PE-fed birds were significantly lower. Indeed, it has been previously reported that polyphenols and flavonoids could suppress lipid peroxidation due to their ROS scavenging properties [58]. MDA is the utmost significant aldehyde formed in the secondary lipid oxidation of polyunsaturated fatty acids (PUFAs) [40]. In our study, MDA levels in breast muscle amongst the dietary groups ranged between 0.38 and 0.58 mg MDA/kg of tissue. Levels between 0.02 and 2.55 mg MDA/kg have been suggested as acceptable limits for non-rancid meat [65]. The obtained results indicate an improvement in breast meat oxidative stability. In agreement with our study, the supplementation of grape seeds and skin meal in broilers’ diet significantly decreased the TBARS levels of thigh meat, indicating the synergetic action of γ- and a-tocopherols for stabilizing lipid peroxidation [66]. Similarly, the inclusion of green tea extract rich in catechins, epigallocatechin-3-gallate, and caffeine in quail diet decreased the MDA concentration in liver and blood plasma [67].

The nutritional requirements and therefore the composition of broilers’ diets have been extensively studied [68]. Thus, there is limited plasticity to implement radical changes and substitutions. Aiming to include feed additives rich in antioxidant compounds but poor in nutritional value, soybean meal and/or soybean oil are usually recruited to balance such substitutions. In our study, the proportion of soybean oil was increased in the GGP group to balance the energy content. Soybean oil is not only rich in PUFAs, predominantly linoleic acid (LA), which are prone to oxidation within the organism, but also their inclusion in concentrate mix could enhance its autooxidation [69]. Thus, the increase in the dietary PUFA level in broilers could induce a cascade of prooxidant incidences [70]. Without narrowing out on soybean oil’s PUFAs in the GGP group, the grape pomace contained a high proportion of ether extract, with the dominant fatty acid being linoleic acid as well. Considering the aforementioned points, there was strong evidence to assume that the increased PUFA inclusion in the GGP group could disturb the oxidative stability of both broilers’ organism and meat [71]. Thus, it is plausible to assume that the high content of polyphenols presented in grape pomace may inhibit a likely induced oxidative burst. Although no significant improvement in the oxidative status of the GGP group was observed, it remains unknown what the PUFA increase would induce in the absence of the antioxidant compounds that were included in grape pomace. Considering the above, the discriminant analysis allocated the PE and WYC groups together since both their polyphenolic compositions and levels were quite comparable. On the contrary, the GGP group was mapped away due to the altered response to dietary treatment attributed to both higher ether extract (PUFA) and polyphenol levels.

Finally, increased mortality (5%; 3 out of 60 birds) in the WYC group was observed during the first week of the starting period, indicating the well-documented stressed transition period of chicks in early life [72]. Even though this proportion tended to differ compared to the CON group, it was within the rational range from an animal scientist’s point of view according to the National Chicken Council [72].

## 5. Conclusions

The exploitation of grape by-products as feed additives appears to be a promising strategy that simultaneously improves waste valorization and supplies animals with bioactive molecules capable of improving animals’ oxidative status and products’ oxidative stability. Considering all the investigated parameters, stem extract and wine lees were found to be promising feed additives in broilers’ diets, focusing on both organisms and meat’s oxidative status improvement. Additional studies are required to investigate the potential of such feed additives towards synthetic antioxidant compounds, their potential to extend animal products’ shelf life and the transfer efficiency of polyphenolic compounds contained in vinification remaining in broilers’ meat.

## Figures and Tables

**Table 1:** Composition (%) of the starting (0–10 day), growing (11–24 day), and finishing (25–42 day) phase of the control (CON), ground grape pomace (GGP), wine lees (rich in yeast cell walls) extract (WYC), and grape stem extract (PE) diets.

Ingredients	Dietary Treatment			
	CON	GGP	WYC	PE
Starter period (Day 1–10)				
Maize	50.52	46.27	50.32	50.42
Soyabean meal	40.89	41.25	40.89	40.89
Soybean oil	4.03	5.39	4.03	4.03
Vitamin and mineral premix ^1^	0.2	0.2	0.2	0.2
Limestone	1.61	1.60	1.61	1.61
NaCl	0.40	0.40	0.40	0.40
Monocalcium phosphate	1.43	1.46	1.43	1.43
Methionine	0.41	0.42	0.41	0.41
Lysine	0.27	0.26	0.27	0.27
Threonine	0.15	0.16	0.15	0.15
Choline	0.09	0.09	0.09	0.09
Ground grape pomace	-	2.5	-	-
Dried wine-making yeast cell walls	-	-	0.2	-
Grape stem phenolic extract included in starch	-	-	-	0.1
Grower period (Day 11–24)				
Maize	53.90	49.92	53.70	53.80
Soyabean meal	37.09	37.40	37.09	37.09
Soybean oil	4.97	6.09	4.97	4.97
Vitamin and mineral premix ^1^	0.2	0.2	0.2	0.2
Limestone	1.47	1.46	1.47	1.47
NaCl	0.4	0.4	0.4	0.4
Monocalcium phosphate	1.22	1.25	1.22	1.22
Methionine	0.36	0.37	0.36	0.36
Lysine	0.20	0.20	0.20	0.20
Threonine	0.11	0.12	0.11	0.11
Choline	0.08	0.09	0.08	0.08
Ground grape pomace	-	2.5	-	-
Dried wine-making yeast cell walls	-	-	0.2	-
Grape stem phenolic extract included in starch	-	-	-	0.1
Finisher period (Day 25–42)				
Maize	59.35	55.40	59.15	59.25
Soyabean meal	31.49	31.79	31.49	31.49
Soybean oil	5.41	6.53	5.41	5.41
Vitamin and mineral premix ^1^	0.2	0.2	0.2	0.2
Limestone	1.34	1.33	1.34	1.34
NaCl	0.40	0.40	0.40	0.40
Monocalcium phosphate	1.09	1.11	1.09	1.09
Methionine	0.34	0.35	0.34	0.34
Lysine	0.21	0.21	0.21	0.21
Threonine	0.08	0.09	0.08	0.08
Choline	0.09	0.09	0.09	0.09
Ground grape pomace	-	2.5	-	-
Dried wine-making yeast cell walls	-	-	0.2	-
Grape stem phenolic extract included in starch	-	-	-	0.1

^1^ Premix supplied per kg of diet: 13,000 IU vitamin A (retinyl acetate), 3500 IU vitamin D3 (cholecalciferol), 70 mg vitamin E (DL-α-tocopheryl acetate), 7 mg vitamin K3, 8.5 mg thiamin, 8 mg riboflavin, 5 mg pyridoxine, 0.020 mg vitamin B12, 50 mg nicotinic acid, 15 mg pantothenic acid, 1.5 mg folic acid, 0.15 mg biotin, 1 mg iodine, 50 mg iron, 75 mg manganese, 15 mg copper, 0.3 mg selenium, 75 mg zinc.

**Table 2:** Composition (%) and calculated analysis of the starting (0–10 day), growing (11–24 day), and finishing (25–42 day) phase of the control (CON), ground grape pomace (GGP), wine lees (rich in yeast cell walls) extract (WYC), and grape stem extract (PE) diets.

Ingredients	Dietary Treatment			
	CON	GGP	WYC	PE
Starter period (Day 1–10)				
Dry matter %	91.5	90.7	90.6	90.5
Ash %	6.7	8	6.6	6.6
Crude protein %	24	23.4	23.0	23.9
Ether extract %	5.7	7	5.8	5.6
Crude fiber %	2.2	2.8	2.5	2.4
ME (Mj/kg)	12.5	12.5	12.5	12.5
Sodium %	0.16	0.16	0.16	0.16
Calcium %	0.96	0.96	0.96	0.96
Phosphorus %	0.48	0.48	0.48	0.48
Lysine %	1.44	1.44	1.44	1.44
Methionine and cysteine	1.08	1.08	1.08	1.08
Threonine %	0.97	0.97	0.97	0.97
Grower period (Day 11–24)				
Dry matter %	91.4	91.3	90.3	89.9
Ash %	6.7	8.8	6.6	6.6
Crude protein %	21.9	21.5	22.0	21.4
Ether extract %	6.6	7.8	6.5	6.4
Crude fiber %	2.3	3	2.5	2.4
ME (Mj/kg)	13	13	13	13
Sodium %	0.16	0.16	0.16	0.16
Calcium %	0.87	0.87	0.87	0.87
Phosphorus %	0.43	0.43	0.43	0.43
Lysine %	1.29	1.29	1.29	1.29
Methionine and cysteine %	0.99	0.99	0.99	0.99
Threonine %	0.88	0.88	0.88	0.88
Finisher period (Day 25–42)				
Dry matter %	90.0	90.0	90.0	91.1
Ash %	6.7	9	6.6	6.6
Crude protein %	19.8	20.4	19.5	19.8
Ether extract %	7.3	8.3	7	7
Crude fiber %	2.3	2.9	2.8	2.5
ME (Mj/kg)	13.4	13.4	13.4	13.4
Sodium %	0.16	0.16	0.16	0.16
Calcium %	0.79	0.79	0.79	0.79
Phosphorus %	0.4	0.4	0.4	0.4
Lysine %	1.16	1.16	1.16	1.16
Methionine and cysteine %	0.91	0.91	0.91	0.91
Threonine %	0.78	0.78	0.78	0.78

**Table 3:** Sequences and relative positions of primers for target genes used in real-time qPCR.

Gene	Sequence	Amplicon bp	Accession No. *	References
*GAPDH*	F: 5′-GCTGGCATTGCACTGAATGAC-3′	113	NM_204305.1	
	R: 5′-CACTCCTTGGATGCCATGT-3′			
*ACTB*	F: 5′-AGCGAACGCCCCCAAAGTTCT-3′	139	NM_205518.1	[31]
	F: 5′-AGCTGGGCTGTTGCCTTCACA-3′			
*CAT*	R: 5′-TGGCGGTAGGAGTCTGGTCT-3′	112	NM_001031215.1	[32]
	R: 5′-GTCCCGTCCGTCAGCCATTT-3′			
*GPX1*	F: 5′-AACCAATTCGGGCACCAG-3′	122	NM_001277853.2	
	R: 5′-CCGTTCACCTCGCACTTCTC-3′			
*GPX2*	F: 5′-GAGCCCAACTTCACCCTGTT-3′	75	NM_001277854.2	
	R: 5′-CTTCAGGTAGGCGAAGACGG-3′			
*SOD1 (CuZn)*	F: 5′-CACTGCATCATTGGCCGTACCA-3′	224	NM_205064.1	[33]
	R: 5′-GCTTGCACACGGAAGAGCAAGT-3′			
*GSTA2*	F: 5′-GCCTGACTTCAGTCCTTGGT-3′	138	XM_015284825.3	[33]
	R: 5′-CCACCGAATTGACTCCATCT-3′			
*NOS2*	F: 5′-AAAGAAAGGGATCAAAGGTGGT-3′	296	NM_204961.1	[34]
	R: 5′-CAAGCATCCTCTTCAAAGTCTG-3′			
*NOX1*	F: 5′-TCATCACTCTGGCGCTCATC-3′	171	XM_040698828.1	
	R: 5′-CCTTCATGCTCTCCTCCGTC-3′			
*NOX2*	F: 5′-TGGTGCGGTTTTGGAGATCA-3′	145	XM_040698636.1	
	R: 5′-GACACTGCTGGGCATTTGAC-3′			
*NOX3*	F: 5′-TTGGAATGGGAGAAGGCCAC-3′	92	XM_040667279.1	
	R: 5′-AGCACCACAGGACTCACAAC-3′			

* Ref Seq: NCBI Reference Sequence database.

**Table 4:** Chemical composition (%) and fatty acid profile of ground grape pomace (GGP).

Ground Grape Pomace GGP	
Chemical composition	
Dry matter %	92.36
Ash %	5.62
Crude protein %	8.00
Ether extract %	9.69
Neutral detergent fiber, NDF %	37.10
Acid detergent fiber, ADF %	34.65
Crude fiber %	24.27
Starch %	1
Ca %	0.15
P %	0.50
Na %	0.01
Fatty acid profile (% of total FA)	
Lauric acid (C12:0)	0.04
Myristic acid (C14:0)	0.14
Stearic acid (C18:0)	4.00
Oleic acid (C18:1 cis-9)	19.33
Linoleic acid (C18:2 n-6 cis)	62.42
α-Linolenic acid (C18:3 n-3)	0.33
γ-Linolenic acid (C18:3 n-6)	0.49
Eicosatrienoic acid (C20:3 n-3)	0.35

**Table 5:** Total Phenolic/Flavonoid/Tannin Content of grape by-products used in broilers’ diets.

	Grape by-Products		
	GGP	WYC	PE
TPC	193.2 ± 33.4	7.3 ± 0.5	10.0 ± 0.9
TFC	50.3 ± 0.8	2.02 ± 0.06	3.73 ± 0.02
TTC	319.5 ± 33.2	10 ± 1	9 ± 2

Concentrations are expressed as mg equivalents (E)/25 g dry material for GGP, as mg E/g starch feed additive from stem extract (PE), and as mg E/2 g dry material for wine lees (WYC). The results were expressed as mg Gallic Acid Equivalents (GAE), mg Quercetin Equivalents (QE), and mg Epicatechin Equivalents (EE) for the three assays, TPC, TFC, and TTC, respectively. All values are means ± standard deviation of three measurements.

**Table 6:** Polyphenolic composition in grape by-products used in broilers’ diets.

	Grape by-Products		
	GGP	PE	WYC
Gallic Acid	6 ± 1	0.080 ± 0.002	0.146 ± 0.008
Caftaric Acid	1.087 ± 0.006	0.164 ± 0.005	0.23 ± 0.02
Procyanidin B1	4.7 ± 0.4	0.215 ± 0.009	0.035 ± 0.002
Epigallocatechin	ND	ND	0.0027 ± 0.0002
Chlorogenic Acid	ND	ND	0.0003 ± 0.0001
Catechin	41 ± 1	0.25 ± 0.01	0.014 ± 0.002
Coutaric Acid	0.35 ± 0.03	0.079 ± 0.003	0.045 ± 0.002
Syringic Acid	0.14 ± 0.03	0.004 ± 0.001	ND
Oenin	0.202 ± 0.008	0.00600 ± 0.00009	0.0499 ± 0.0009
Procyanidin B2	14.3 ± 0.3	0.0025 ± 0.0007	0.045 ± 0.004
Fertaric Acid	0.25 ± 0.01	0.0085 ± 0.0001	0.056 ± 0.002
Epicatechin	24.460 ± 0.008	0.009 ± 0.006	0.132 ± 0.006
Caffeic Acid	ND	ND	0.117 ± 0.002
Epigallocatechin Gallate	0.0590 ± 0.0004	0.009 ± 0.004	0.015 ± 0.001
Polydatin	ND	Tr	0.096 ± 0.008
Quercetin-3-b-D-glucoside	2.9 ± 0.2	0.0763 ± 0.0009	0.274 ± 0.004
Epicatechin Gallate	0.83 ± 0.05	0.0132 ± 0.0009	0.0169 ± 0.0005
Procyanidin A2	ND	ND	ND
Rutin	ND	ND	0.0011 ± 0.0001
*p*-Coumaric Acid	0.040 ± 0.009	ND	0.175 ± 0.002
Ferulic Acid	0.146 ± 0.007	Tr	0.0076 ± 0.0003
Sinapic Acid	ND	ND	Tr
*trans*-Resveratrol	0.0374 ± 0.005	0.011 ± 0.006	0.007 ± 0.001
Myricetin	ND	ND	0.065 ± 0.001
*o*-Coumaric Acid	ND	ND	ND
Quercetin	2.0 ± 0.2	0.0230 ± 0.0007	0.2789 ± 0.0006
Apigenin	0.5 ± 0.2	0.004 ± 0.002	ND
Kaempferol	0.16 ± 0.02	ND	0.090 ± 0.002
Isorhamnetin	0.080 ± 0.003	ND	0.1297 ± 0.0005

ND = Not Detected; Tr = traces; Concentrations are expressed as ng/25 g dry material for grape pomace (GGP), as ng/g starch feed additive from stem extract (PE), and as ng/2 g dry material for wine lees (WYC). All values are means ± standard deviation of three measurements.

**Table 7:** Broiler’s growth performance on starter, grower, finisher, and overall experimental period among the four dietary treatments (Control; CON, ground grape pomace; GGP, dried wine lees; WYC, and extract from grape stems included in soluble starch; PE).

	Dietary Treatment					
	CON	GGP	WYC	PE	SEM	Effect
Initial BW (g/broiler)	44.08	44.08	44.79	44.50	0.54	NS
Overall experimental period						
Final BW (g/broiler)	2975.8	2981.9	3004.9	3103.7	61.81	NS
FI (g/broiler)	4204.1	4195.5	4199.3	4414.7	77.43	NS
FCR	1.43	1.43	1.42	1.44	0.02	NS
Mortality % (broilers)	0 (0/60)	3.3 (2/60)	5.0 (3/60)	1.7 (1/60)	-	NS

Final BW: final body weight; FI: feed intake; FCR: feed conversion ratio (g feed/g gain); SEM: pooled standard error of means.

## Data Availability

All data are presented within the article.
